# Multicenter study demonstrates radiomic features derived from magnetic resonance perfusion images identify pseudoprogression in glioblastoma

**DOI:** 10.1038/s41467-019-11007-0

**Published:** 2019-07-18

**Authors:** Nabil Elshafeey, Aikaterini Kotrotsou, Ahmed Hassan, Nancy Elshafei, Islam Hassan, Sara Ahmed, Srishti Abrol, Anand Agarwal, Kamel El Salek, Samuel Bergamaschi, Jay Acharya, Fanny E. Moron, Meng Law, Gregory N. Fuller, Jason T. Huse, Pascal O. Zinn, Rivka R. Colen

**Affiliations:** 1 Department of Diagnostic Radiology, The University of Texas MD Anderson Cancer Center, Houston, TX 77030 USA; 2 Department of Cancer Systems Imaging, The University of Texas MD Anderson Cancer Center, Houston, TX 77054 USA; 3 Department of Restorative and Dental Materials, National Research Centre, Cairo, 12622 Egypt; 4 Department of Radiology, University of Southern California, Keck School of Medicine, Los Angeles, CA 90033 USA; 5 Department of Radiology, Baylor College of Medicine, Houston, TX 77030 USA; 6 Alfred Health & Monash University, Melbourne, 3004 Australia; 7 Department of Pathology, Anatomical and Translational Molecular Pathology, The University of Texas MD Anderson Cancer Center, Houston, TX 77030 USA; 8 Department of Neurosurgery, Baylor College of Medicine, Houston, TX 77030 USA; 9 Department of Neurological Surgery, University of Pittsburgh, Pittsburgh, PA 15213 USA; 10 UPMC Hillman Cancer Center, Pittsburgh, PA 15232 USA; 11 Department of Radiology, University of Pittsburgh, Pittsburgh, PA 15213 USA

**Keywords:** Cancer, Cancer imaging

## Abstract

Pseudoprogression (PsP) is a diagnostic clinical dilemma in cancer. In this study, we retrospectively analyse glioblastoma patients, and using their dynamic susceptibility contrast and dynamic contrast-enhanced perfusion MRI images we build a classifier using radiomic features obtained from both Ktrans and rCBV maps coupled with support vector machines. We achieve an accuracy of 90.82% (area under the curve (AUC) = 89.10%, sensitivity = 91.36%, 67 specificity = 88.24%, *p* = 0.017) in differentiating between pseudoprogression (PsP) and progressive disease (PD). The diagnostic performances of the models built using radiomic features from Ktrans and rCBV separately were equally high (Ktrans: AUC = 94%, 69 *p* = 0.012; rCBV: AUC = 89.8%, *p* = 0.004). Thus, this MR perfusion-based radiomic model demonstrates high accuracy, sensitivity and specificity in discriminating PsP from PD, thus provides a reliable alternative for noninvasive identification of PsP versus PD at the time of clinical/radiologic question. This study also illustrates the successful application of radiomic analysis as an advanced processing step on different MR perfusion maps.

## Abstract (web summary)

MRI scans of glioblastoma patients can be misleading and some patients appear to show features of progressive disease although they respond to treatment. Here, the authors use MRI images of progressive disease or pseudoprogression and build a classifier using machine learning to distinguish the two.

## Introduction

The current standard of treatment for glioblastoma is the combination of maximal safe resection, radiation, and chemotherapy; this paradigm was shown to prolong the median overall survival to 14.6 months^1,2^. Immunotherapeutic agents, which utilize the body’s innate immune responses to kill cancerous cells, have demonstrated success in preclinical trials^3–5^. However, during treatment, the size of the tumor often increases and/or new inflammatory lesions appear. These transient changes typically stabilize or subside without further treatment^6^, but they are often difficult to distinguish from progressive disease (PD). This PD-mimicking phenomenon is called pseudoprogression (PsP). Patients with PsP have longer overall survival and are less likely to exhibit signs and symptoms of neurological deterioration than those with PD^7,8^.

Early discrimination of PsP from PD is a clinical challenge. Surgical biopsy is used in current clinical practice as a standard procedure for diagnosis of recurrent or residual disease, but biopsy is not only highly invasive but also limited in accuracy depending on the biopsy site, type of resection, and lesion heterogeneity^9^. Furthermore, biopsy is not feasible in every glioblastoma patient who has enhancement on post-treatment imaging^9^.

Conventional magnetic resonance imaging (MRI) and the Response Assessment in Neuro-Oncology (RANO) criteria are used as an alternative to surgical biopsy for distinguishing PsP from PD, but the diagnostic performance of this evaluation varies considerably^10^. By RANO criteria, patients with clinical deterioration, a new lesion, or an increase in existing tumor size (≥25% increase in the sum of products of perpendicular diameters) on restaging MR scans are defined as having PD, while those with an increase <25% or a decrease of <50% in the sum of products of perpendicular diameters of enhancing lesion are considered to have stable disease, as shown in Supplementary Table 1^10^. Immunotherapy RANO (iRANO) criteria were devised as an update to RANO to monitor patients undergoing immunotherapy^11^. According to the iRANO criteria, patients with evidence of PD (per RANO) within 6 months of immunotherapy who do not develop considerable neurological decline should continue on the current therapy and undergo follow-up imaging in 3 months for confirmation of PD^11^. The drawback of this watchful waiting is that patients with PD will continue on an ineffective therapy, incurring the unnecessary risks of potential toxicity and delay in switching to more effective therapy; furthermore, failure to correctly identify PsP may result in premature discontinuation of an effective treatment^10,11^.

Recently, there have been numerous efforts using MR perfusion to differentiate PD from PsP^12–14^. MR perfusion, which can assess the vascular properties of the post-treatment enhancing lesions, calculates changes in blood volume, blood flow, and vessel wall permeability. These are important characteristics of tumor vessels^15,16^. However, lack of standardization in the post-processing steps of MR perfusion studies has led to a discrepancy in the reported cut-off values determining post-treatment changes from PD and corresponding sensitivity and specificity^17,18^. Therefore, there is a need for an accurate noninvasive quantitative tool to differentiate between PD and pseudoprogression at the time of detection of a brain MRI questionable lesion.

Radiomic analysis is a new automated, high-throughput method that quantifies the tumor phenotype at a microscale level (voxel/pixel level) by using thousands of image-based features obtained by histogram and texture analysis^19–21^. Unlike surgical biopsy, radiomic analysis assesses the entire three-dimensional tumor inclusive of spatial heterogeneity^22,23^. Prior studies have employed radiomic analysis on MR perfusion for predicting the underlying glioblastoma molecular phenotype^20,24–26^ and patient outcomes^24,25^ as well as distinguishing benign and malignant breast lesions^27^. However, to date, no multicenter investigations on the use of MR perfusion-based radiomic analysis to distinguish histologically-proven PsP from PD in glioblastoma have been reported.

In this multicenter study, we seek to determine the ability of MR perfusion-based radiomics to discriminate PsP from PD in glioblastoma patients. We evaluated 98 patients with pathologically-proven glioblastoma to identify the MR perfusion-based radiomic signatures of PsP and PD; we then combined these signatures to develop a noninvasive predictive model to robustly differentiate PsP from PD in the clinical setting. With an inclusive and diverse multi-institutional cohort, this pioneering study seeks to distinguish PD from PsP through MR perfusion-based radiomic analysis.

## Results

### Patient characteristics

We investigated a total of 98 patients from 3 institutions who had histopathologic evidence of PD (*n* = 76; 77.6%) or PsP (*n* = 22; 22.4%; Table 1). Using Mann-Whiteney test for non-prametric data and independent sample *t* test for parametric data, there were no statistical significant difference between the two groups in terms of age, sex, or K_trans_ volume (*p* > 0.05). However, the exctracted VOI of rCBV using 3D slicer were differed significantly between PsP and PD groups (1126.91 mm^3^ and 1369.44 mm^3^, respectively; *p* = 0.010).

**Table 1:** Baseline selected demographic and clinical characteristics of patients with Pseudo progression/Glioblastoma grade IV (*N* = 98)

Characteristic	Pseudoprogression *N* = 22	Glioblastoma grade IV *N* = 76
Age, years (SD)	53.50 (14.88)	49.37 (12.97)
Sex, male, *N* (%)	15 (68.2%)	42 (54.7%)
K_trans_ volume, mm^3^ (SD)	3166.99 (4618.17)	4591.56 (6233.76)
rCBV volume, mm^3^ (SD)	1126.91 (2771.41)	1396.44 (2245.20)
Surgical type:		
Total resection, *N* (%)	19(86.4%)	45(59.2%)
Sub-total resection, *N* (%)	3(13.6%)	29(38.2%)
Biopsy, *N* (%)	0(0%)	2(2.6%)
Molecular status:		
*MGMT*		
Methylated, *N* (%)	3(13.7%)	3(3.9%)
Unmethylated, *N* (%)	1(4.5%)	10(13.2%)
Non tested, *N* (%)	18(81.8%)	63(82.9%)
*IDH*		
Positive, *N* (%)	2(9%)	7(9.2%)
Negative, *N* (%)	5(22.7%)	16(21%)
Non tested, *N* (%)	15(68.3%)	53(69.8%)
Radio-therapy time, days (SD)**	54.21429(36.59)	56(22.24)
Time after RT to PD/PSP, days(SD)***	779.92(766.59)	664.9857 (854.85)
Chemotherapy treatment (Temozolomide), *N* (%)	22 (100%)	76 (100%)

*rCBV* relative cerebral blood volume, *SD* standard deviation

*, Significant difference

**, 5 PSP and 8 PD with no available data

***, 2 PSP and 5 PD with no available data

### Predictive value of perfusion parameters analysis

Perfusion parameter analysis showed that neither K_trans_ nor rCBV was able to predict PsP from PD groups, and there was no statistically significant difference between the PsP and PD groups (Mann-Whitney test for non-parametric data; Supplementary Table 4).

### Performance of the radiomic model

**Fig. 1:**
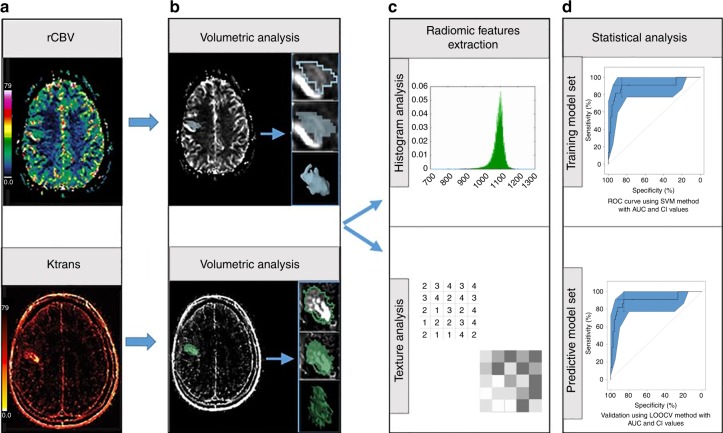
Image post-processing radiomic workflow. **a** relative cerebral blood volume (rCBV) and K_trans_ maps of perfusion MRI are acquired. **b** Segmentation of the region of interest using 3D slicer software. **c** Radiomic feature extraction from the whole tumor volume. **d** Statistical analysis: radiomic and clinical features are analyzed to determince their diagnostic and predictive values

To determine the capacity of radiomic features to distinguish PsP from PD, we performed an integrative analysis assessing the predictive performance of perfusion radiomic features. The image post-processing workflow is shown in Fig. 1. We first ranked features based on their relevance to the outcome and within-feature redundancy using the MRMR feature selection technique. Subsequently, SVM with linear kernel and C5.0 models were constructed using the features selected by the MRMR analysis. Finally, the diagnostic performance of the models was evaluated using LOOCV for the SVM model and 10-fold cross-validation for the C5.0 model.

**Fig. 2:**
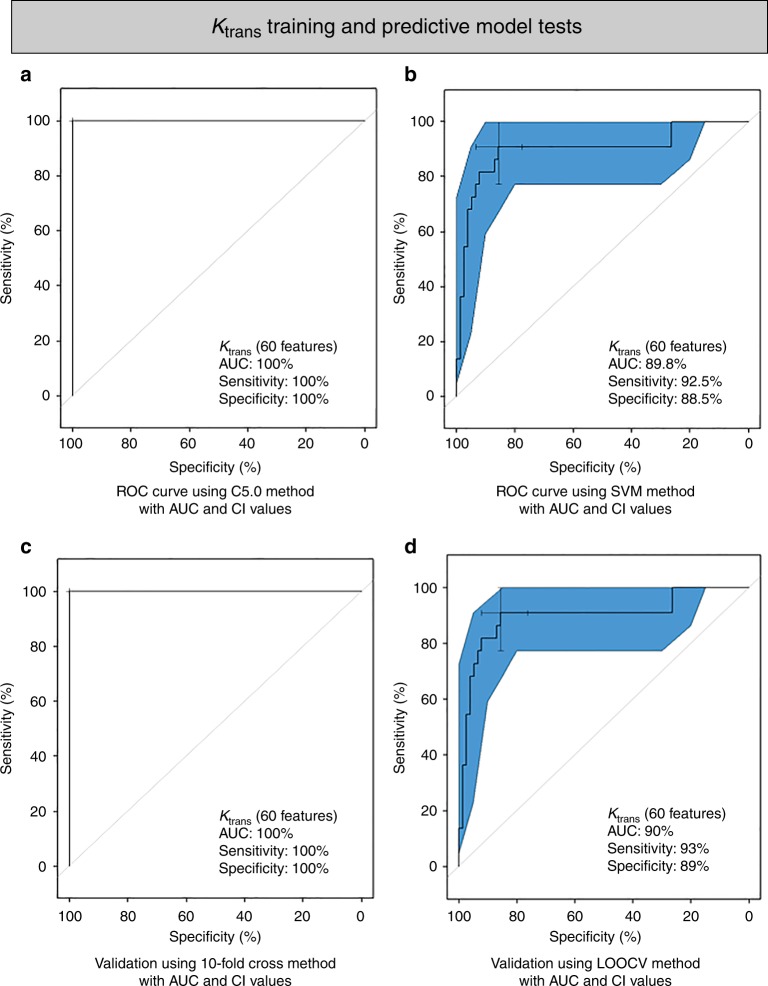
Model building and evaluation using the selected K_trans_ features (60 features). **a**, **b** ROC curve depicts the predictive model building using C5.0 (*P*-value 1.512e−11) and SVM methods (*P*-value 0.003744) respectively. **c**, **d** 10-fold cross-validation ROC curve (*P*-value 1.512e−11) and Leave-One-Out Cross-Validation (LOOCV) ROC curve (*P*-value 0.004) depicts the performance of the model

In the analysis using K_trans_ maps, the top 60 features (as ranked by the MRMR feature selection technique) robustly differentiate between PsP and PD. As shown in Fig. 2, selected radiomic features significantly discriminated between PsP and PD using the C5.0 method (AUC 100%, sensitivity 100%, specificity 100%, *p-*value 1.512e−11 one sided binomial test). Similar results were obtained using the SVM method (AUC 89.8%, sensitivity 92.5%, specificity 88.89%, *p-*value 0.003744 one sided binomial test) (Fig. 2). We then validated the predictive models using LOOCV (AUC 90%, sensitivity 93%, specificity 89%, *p-*value 0.004 one sided binomial test, Fig. 2) and 10-fold cross-validation (AUC 100%, sensitivity 100%, specificity 100%, *p-*value 1.512e−11 one sided binomial test, Fig. 2). Significant features used in the models are shown in Supplementary Table 5.

**Fig. 3:**
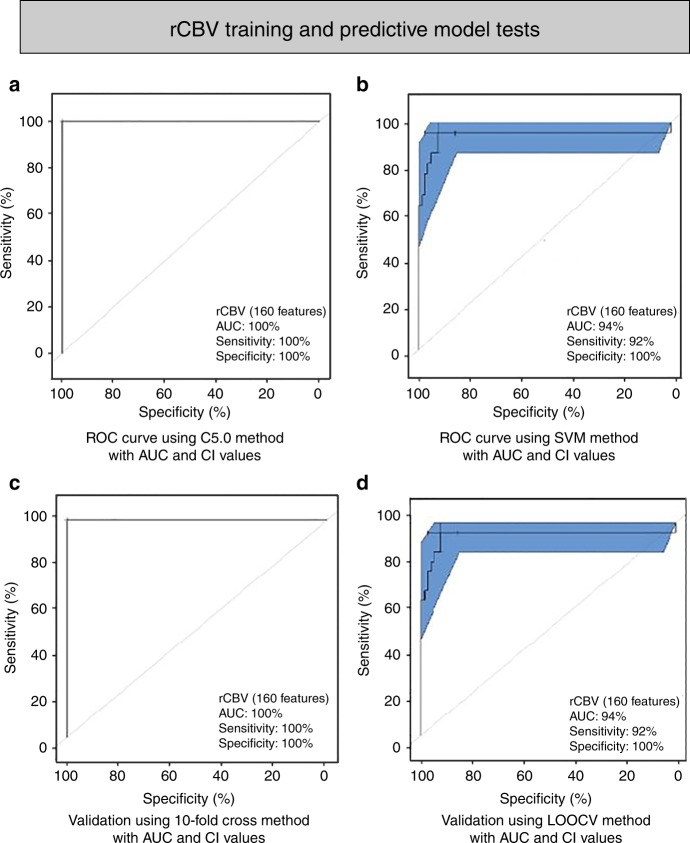
Model building and evaluation using the selected rCBV features (160 features). **a**, **b** ROC curve depicts the predictive model building using C5.0 (*P*-value 1.512e−11) and SVM methods (*P*-value 0.012) respectively. **c**, **d** 10-fold cross-validation ROC curve (*P*-value 1.512e−11) and Leave-One-Out Cross-Validation (LOOCV) ROC curve (*P*-value 0.012) depicts the performance of the model

In the analysis using rCBV maps, the top 160 features (as identified by the MRMR feature selection technique) achieved the highest predictive accuracy in differentiating between PsP and PD. Significant features used in the models are shown in Supplementary Table 6. Radiomic models built using SVM significantly distinguished PsP and PD (AUC 94%, sensitivity 92%, specificity 100%, *p-*value 0.012 one-sided binomial test); similar results were obtained using the C5.0 classification (AUC 100%, sensitivity 100%, specificity 100%, *p-*value 1.512e−11 one-sided binomial test). Validation using LOOCV (AUC 94%, sensitivity 92%, specificity 100%, *p-*value 0.012 one-sided binomial test, Fig. 3) and 10-fold cross-validation (AUC 100%, sensitivity 100%, specificity 100%, *p-*value 1.512e−11 one-sided binomial test, Fig. 3) demonstrated to be highly statistically significant.

**Fig. 4:**
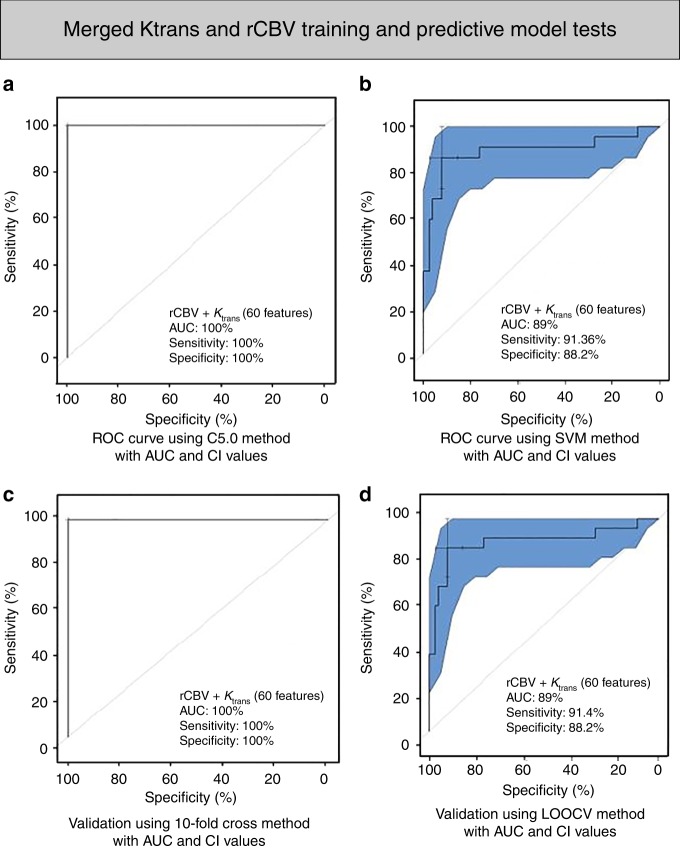
Model building and evaluation using the selected merged K_trans_ and rCBV features (60 features). **a**, **b** ROC curve depicts the predictive model building using C5.0 (*P*-value 1.512e−11) and SVM methods (*P*-value 0.017), respectively. **c**, **d** 10-fold cross-validation ROC curve (*P*-value 1.512e−11) and Leave-One-Out Cross-Validation (LOOCV) ROC curve (*P*-value 0.02) depicts the performance of the model

To determine the added value of combining the radiomic features obtained from both K_trans_ and rCBV pharmacokinetic maps in predicting PsP from PD, we first ranked all 620 features identified by the MRMR technique and built further predictive models. Using 60 radiomic features (Supplementary Table 7) coupled with SVM, we achieved an accuracy of 90.82% (AUC 89.10%, sensitivity 91.36%, specificity 88.24%, *p-*value 0.017 one-sided binomial test); similar results were obtained using C5.0 (AUC 100%, sensitivity 100%, specificity 100%, *p-*value 1.512e−11 one-sided binomial test). Differences identified by LOOCV predictive modeling (AUC 89%, sensitivity 91.4%, specificity 88%, *p-*value 0.02 one-sided binomial test, Fig. 4) and 10-fold cross-validation (AUC 100%, sensitivity 100%, specificity 100%, *p-*value 1.512e−11 one-sided binomial test, Fig. 4) were both statistically significant.

As the image acquisition parameters were distinct between different institutions, we sought to determine the scalability and generalizability of our radiomics analytical technique. Towards this end, we performed sub-analysis on the 63 MDACC patients, we achieved a high accuracy, sensitivity, and specificity to differentiate between PSP and PD using the three different extracted radiomic models obtained from Ktrans, rCBV and combined pharmacokinetic maps as we demonstrated in Supplementary Figs 1–3.

To assess the predictive power of the extracted radiomic features obtained from Ktrans, rCBV, and combined pharmacokinetic maps, we collated an independent prospective dataset of seven pathologically proven MDACC patients (Supplementary Table 2). Using the model built with whole dataset on the 98 patients as well as models built with cross-validation technique (LOOCV and 10-fold CV) for both C5.0 and SVM methods, the prediction results showed that the 7 new prospective patients were correctly classified as PD patients with high probability percentage as mentioned in Supplementary Tables 8–10.

## Discussion

In this study, we evaluated the ability of MR perfusion-based radiomics to non-invasively discriminate between PsP and PD in glioblastoma patients at the time of clinical/radiologic question. We demonstrated that, although PsP and PD have similar radiographic appearances, they harbor distinct radiomic information that is hidden within MR perfusion images and can be extracted and used to build a clinically-relevant predictive model that discriminates PsP from PD.

Evaluation of post-treatment changes in glioblastoma patients using conventional MRI remains a challenge for clinicians and radiologist, as post-treatment MR images demonstrate confounding appearances^28–30^. Advanced MRI techniques, including perfusion MRI, have been proposed as alternatives to current imaging assessment criteria; however, reported cut-off values determining post-treatment changes versus PD vary widely across different studies. In the meta-analysis by Wan et al., they demonstrated substantial discrepancy between the cut-off values (see Wan et al.; Table 1)^18^, which they attributed to the differences in the acquisition parameters of DSC and DCE among different institutions. Further, the reported accuracy, sensitivity, and specificity of the perfusion studies are not clinically reliable^17^.

In our patient cohort, ROI-based perfusion parameter analysis using K_trans_ and rCBV pharmacokinetic maps alone did not detect any differences between PsP and PD patient groups (Supplementary Table 4). However, our proposed radiomic model achieved high accuracy, sensitivity, and specificity in differentiating between PsP from PD compared to previous methods at the time of the clinical question of PsP versus PD; therefore, radiomics can be used by radiologists and oncologists for clinical decision-making (Figs. 2–4).

The radiomic model used in this study and proposed for ongoing clinical applications was built using imaging data from a multi-institutional cohort of patients for whom histopathologic confirmation of PsP or PD was available. The model demonstrated high discriminatory power, with AUCs greater than 89% (Figs. 2–4). Despite differences in the MR acquisition parameters of DSC and DCE imaging among the institutions (Supplementary Table 3), the radiomics predictive model demonstrated robust performance. Additionally, the discriminatory power was highly robust for the individual pharmacokinetic maps and after fusing the features from both the K_trans_ and rCBV maps. These results suggest that our radiomic model is well-calibrated and can be directly applied in any clinical setting without the need to adjust the acquisition protocol or include both DSC and DCE sequences.

Further, the majority of the features included in our models are second-order (Supplementary Tables 5–7); of the key features selected (60 for K_trans_, 160 for rCBV and 60 for fused model), the number of second-order features was 54 (90%) for the K_trans_-based model (Supplementary Table 5), 150 (94%) for the rCBV-based model (Supplementary Table 6), and 54 (90%) for the fused model (Supplementary Table 7). This finding is concordant with the fact that histogram features depict the data distribution within a selected region, and therefore their value is directly affected by the differences in acquisition parameters. Conversely, second-order features capture the inter-relationship between neighboring voxels, and thus are not sensitive to the absolute data value. Hence, by incorporating second-order radiomic features, the model is more robust and ‘immune’ to the MR acquisition parameters.

The robustness of our extracted radiomic features is further demonstrated by the similarity in calculated AUC, sensitivity, and specificity for the two classification algorithms investigated: (i) SVM with linear kernel and (ii) C5.0. A previous study investigated the performance and stability of various feature selection and classifier methods in radiomic applications^31^. They reported variable accuracy for different combinations of feature selection and classifier methods. Although evaluation of classifier methods and feature selection techniques was beyond the scope of this work, our findings indicate that our extracted radiomic features were stable and performed equally well independent of the classifier method^31^.

Further, the key features selected for both K_trans_- and rCBV-based models were entropy, sum of squares, and autocorrelation (Supplementary Tables 5–7). These features hold information about the spatial heterogeneity of the studied region and are more sensitive when assessing tumor microenvironment and the presence of tumor cells. The finidngs of the present study confirmed and extended previous work by Zinn et al. who showed that MRI features (Diffsuion and radiomic features) capture spatial heterogeneity^25,32,33^. Our results show that patients with PD have high entropy and sum of squares and low autocorrelation compared to PsP patients. Thus, selected volumes in PD patients were characterized by non-uniformity and increased complexity compared to PsP patients. This may reflect the heterogeneity seen microscopically^34–36^.

Limitations of the current study mostly result from the requirement for histopathologic confirmation of PsP or PD. First, the percentage of patients with PsP is low, and our study design did not include patients for whom histopathologic confirmation was not available. Because biopsy is an invasive procedure and clinicians usually prefer a watchful approach over biopsy, it is expected that only a small fraction of patient who have undergone biopsy will have PsP. Second, we performed semi-automated segmentation of the VOI, which is a time-consuming process and also requires an in-depth assessment of inter-rater variability and stability of the extracted radiomic features. A user-friendly tool that performs reliable automatic segmentations is needed, as it would allow for easy translation of the radiomic analysis in the clinical setting. Additionally, being a retrospective study, important information, such as molecular and genetic characteristics, was not available to review. For example, the contribution of MGMT methylation and *IDH1/2* mutations, was not investigated in this work, since this information was not available for the majority of the studied population. Finally, the total number of patients included in the study was relatively small and did not allow for a large validation prospective dataset using as a separate cohort. A much larger study with a larger patient cohort that will allow independent validation is needed; further the prospective validation component is currently performed in our institution.

In summary, our study presents a radiomic model based on MR perfusion data for noninvasive, individualized prediction of PsP in glioblastoma patients at the point-of-care (at the time of the clinical question). We demonstrate the high predictive accuracy of radiomics in differentiating PsP from PD. Our radiomic model can be easily integrated into the clinical setting, as it is a post-processing approach that does not require changes in the current imaging protocol and will allow clinicians to make more informed decisions for optimal patient care. After outlining the VOI on DCE and DSC maps, the model will indicate whether the patient has PsP or PD. Our radiomic model will complement the methods now available to clinicians by offering a comprehensive evaluation of the imaging data. We expect that it will dramatically decrease the number of invasive procedures performed to confirm the absence of tumor in patients; Most importantly, our radiomics method can help ensure that patients with PsP will continue on an effective treatment or discontinue an ineffective treatment without the need for delays caused by watchful waiting. Conversely, early identification of PD will allow the prompt transition to a more effective treatment.

## Methods

### Study population

This retrospective multi-institutional clinical study was conducted in compliance with U.S. Health Insurance Portability and Accountability Act (HIPAA) regulations. All necessary institutional ethics review approvals were obtained by the participating institutions (The University of Texas MD Anderson Cancer Center, Baylor College of Medicine, and University of Southern California Keck School of Medicine).

A total of 651 patients with histopathologic diagnosis of GBM and an enhancing lesion on MRI that may indicate PD or treatment-related changes were received from three institutions; The University of Texas MD Anderson Cancer Center (MDACC) (598 patients), Baylor College of Medicine (BCM) (13 patients), and University of Southern California (USC) Keck School of Medicine (40 patients). A total of 98 patients were included in the final analysis based on the following inclusion criteria: (1) minimum age of 18 years; (2) enhancing lesion on MRI that may indicate PD or treatment-related changes; (3) conventional and advanced MRI, including dynamic susceptibility contrast (DSC) and dynamic contrast-enhanced (DCE) perfusion imaging; (4) pathologic confirmation of PD or PsP within 3 months after detection of a questionable enhancing lesion on MRI (time of question); (5) adequate image quality with no artifact affecting the radiomic analysis. 553 patients did not meet the inclusion criteria and therefore excluded. 8 patients (1 BCM, 7 USC) did not have dynamic susceptibility contrast-enhanced T2*-weighted (DSC) and dynamic contrast-enhanced (DCE) perfusion; 85 patients (82 MDACC, 2 BCM, and 1USC) were excluded due to poor quality of imaging/perfusion data; 460 (453 MDACC, 4 USC, and 3 BCM) were excluded due to absence of histopathological confirmation of PsP versus PD.

The clinical, radiologic, and pathologic data were retrieved from the medical records. Our final study cohort consisted of: 63 (50PD and 13PSP) patients from MDACC, 7 (6PD and 1PSP) patients from BCM, and 28 (21PD and 7PSP) patients from USC. Based on the pathologic diagnosis, 76 (77.6%) patients had PD and 22 (22.4%) patients had treatment-related changes considered to be PsP. Detailed demographic, clinical characteristics and treatment information is presented in Table 1.

For further validation of our results, a prospective study of 7 MDACC patients with pathologically proven PD were included using the same aforementioned inclusion and exclusion criteria. (Supplementary Table 2 showed the demographic, clinical and pathological data)

### Histopathologic evaluation

All patients had undergone biopsy or surgical resection, and resected entire tumor/tissue block specimens were histopathologically examined. The pathology status as PD or PSP was decided and reviewed in consensus by two experienced board-certified pathologists (J.T.H. and G.N.F.) blinded to the MRI data. PD was defined as samples with either solely recurrent GBM tissue or mixture of radiation necrosis and recurrent GBM, while PSP was determined by the presence of radiation necrosis only or mixture between radiation necrosis and reactive gliosis.

### MRI acquisitions and imaging post processing

For conventional MRI acquisition, images were acquired using a 1.5 or 3.0 Tesla MR Scanner. The MRI protocol for MDACC included an axial T1-weighted sequence (repetition time [TR], 700 ms; echo time [TE], 12 ms; slice thickness, 5 mm; acquisition matrix 352 × 224), an axial fluid attenuation inversion recovery (FLAIR) sequence (TR, 10,000 ms; TE, 140 ms; slice thickness, 5 mm; acquisition matrix, 256 × 256), and an axial post-contrast T1-weighted sequence acquired 5 min after the contrast injection (TR, 750 ms; TE, 13 ms; slice thickness, 5 mm; acquisition matrix, 384 × 256). The MRI protocol for USC included an axial T1-weighted sequence (TR, 600 ms; TE, 8 ms; slice thickness, 5 mm; acquisition matrix, 320 × 192), an axial FLAIR sequence (TR, 8000 ms; TE, 151.2 ms; slice thickness, 5 mm; acquisition matrix, 320 × 224), and an axial post-contrast T1-weighted sequence acquired 5 min after the contrast injection (TR, 615 ms; TE, 17 ms; slice thickness, 5 mm; acquisition matrix, 320 × 192). The MRI protocol for BCM included an axial T1-weighted sequence (TR, 750 ms; TE, 11 ms; slice thickness, 5 mm; acquisition matrix, 384 × 192), an axial FLAIR sequence (TR, 2541 ms; TE, 16.4 ms; slice thickness, 5 mm; acquisition matrix, 384 × 192), and an axial post-contrast T1-weighted sequence acquired 5 min after the contrast injection (TR, 750 ms; TE, 11 ms; slice thickness, 5 mm; acquisition matrix, 384 × 192). while for advanced MRI acquisition: The parameters of both DCE and DSC for each institution are shown in Supplementary Table 3. Pre-contrast T1 mapping images were acquired with six flip angles (2, 5, 10, 15, 20, and 25 degrees). Images were acquired before and after injection of passively targeted gadolinium-diethylenetriamine penta-acetic acid (0.1 mmol per kg).

The advanced imaging analysis and pharmacokinetic map calculation were performed using Nordic ICE (NordicNeuroLab, Bergen, Norway). Images were segmented using 3D Slicer (version 4.3.1, https://www.slicer.org), an open-source software program widely used for image visualization and segmentation^37,38^. The image post-processing workflow is shown in Fig. 1.

DCE data were analyzed by using Nordic ICE, using the dual compartment modified Tofts and Kermode pharmacokinetic model as described elsewhere^39,40^. We manually selected the arterial input function in the middle cerebral artery located on the ipsilateral side, then the pharmacokinetic K_trans_ parameter (reflects local blood flow, endothelial permeability, endothelial surface area, proportional blood volume within a given voxel) was obtained^41^. For DSC analysis, the arterial input function was manually selected in the middle cerebral artery located on the ipsilateral side. Using the integrated DSC module, which incorporates a relative cerebral blood volume (rCBV) leakage-correction algorithm and manual noise thresholding, we obtained the amount of blood in a given volume of tissue expressed as mL per 100 mL of tissue^41^.

### Perfusion parametric maps analysis

We performed a perfusion parameter analysis to determine whether perfusion parameters can differentiate between PD and PsP. Both capillary permeability (K_trans_) and leakage-corrected rCBV maps were rigidly registered based on mutual information with the post-contrast T1WI using Nordic ICE; the resulting registrations were visually inspected to ensure adequate alignment. Subsequently, circular regions-of-interest (ROIs) with an area of 10.55 mm^2^ were manually delineated on the area with the highest parameter value for each parameter map, ensuring that necrotic areas and large blood vessels were excluded. The mean values were extracted from the ROIs for each parameter map. To reduce sampling error, four ROIs were selected and the average of the mean values was obtained^42^. This method has been demonstrated to provide the optimal interobserver and intra-observer reproducibility^43^.

### Quantitative radiomic analysis and model building

Post-contrast T1WI were coregistered with K_trans_ and rCBV maps to ensure appropriate tumor delineation; cystic, necrotic regions and intralesional macrovessels were excluded. The coregistered images for both K_trans_ and leakage-corrected rCBV maps were semi-automatically segmented by an experienced user (N.E., 4 years experience) and manually reviewed slice-by-slice by an experienced board-certified radiologist (R.R.C., 9 years experience), both of whom were blinded to histopathologic assessment. Three-dimensional volumes of interest (VOIs) were subsequently extracted for further analysis. A total of 310 radiomic features were extracted from each pharmaocokinetic parametric map, consisting of 10 histogram-based features^44^ and 300 s-order Haralick features^45–47^. Accounting for the K_trans_ and leakage-corrected rCBV maps analyzed in this study, a total of 620 radiomic features were obtained per patient^26,48,49^. Radiomic analysis was performed using our in-house developed software implemented in MATLAB (version 2017b; MathWorks Inc; Natick, MA).

Histogram-based features describe the K_trans_ and rCBV values distribution within the VOI; the features obtained were minimum, maximum, mean, standard deviation, skewness, kurtosis, and the percentiles 1%, 5%, 95%, and 99%. The second-order Haralick features were computed from the gray-level co-occurrence matrix (GLCM) after requantization of the image gray levels; the gray levels implemented in this analysis were *8, 16, 32, 64*, and *256*. The second-order Haralick features describe mathematical relationships between co-occurring voxels separated by a given distance in a specific direction. For the purposes of this analysis, we investigated a distance of 1 voxel and 4 in-plane directions. Twenty GLCM-based features were obtained per gray level: autocorrelation, contrast, correlation, cluster shade, cluster prominence, dissimilarity, energy, entropy, homogeneity, maximum probability, variance, sum average, sum variance, sum entropy, difference variance, difference entropy, information measure of correlation 1, information measure of correlation 2, inverse difference moment, and normalized inverse difference moment. Rotation invariant measures of the features were obtained by calculating the average, range, and angular variance of the features across the four in-plane directions. Taking into account the number of gray levels and the number of rotation invariant measures, a total of 300 were calculated per map. Fig. 1 shows the pipeline used to get the radiomic perfusion model.

To investigate the predictive performance of extracted radiomic features in discriminating between PD and PsP (outcome), we first identified the radiomic features (feature set) that are associated with outcome. To minimize irrelevant and redundant radiomic features, we used the Maximum Relevance Minimum Redundancy (MRMR) feature selection technique; MRMR ranks the features based on maximizing the relevance and avoiding excess redundancy^50^. The MRMR approach was implemented using the mRMRe R package.

Different sizes of feature sets were investigated to ensure selection of the fetaureset size with best prediction accuracy. We started with the MRMR-ranked 310 features and evaluated different featureset sizes ranging from 310 to 20 in steps of 10.

MRMR-ranked radiomic features were used for classification and model building. Two different supervised learning algorithms were used in this analysis for classification and predictive model building: Support Vector Machine (SVM) and decision tree algorithm C5.0. In the C5.0 algorithm, a decision tree is used while modeling the classification process^51^. SVM is a supervised machine learning algorithm that is used for solving classification problems by transforming the feature space to a higher dimension space so that a separating hyperplane maximizes the distance between the two classifiers^52^. To increase model accuracy in our analysis, we tested the classifier in SVM with three different kernels (linear, polynomial, and radial basis)^53^.

We used leave-one-out cross-validation (LOOCV) and k-fold cross-validation (k = 10) to evaluate the accuracy, sensitivity, and specificity of the models^54^. Receiver Operating Characteristic (ROC) curves were plotted; and the area under the curve (AUC), accuracy, sensitivity, specificity, positive predictive value (PPV), negative predictive value, and *p*-value were reported for each cross-validation and prediction output.

### Statistical analysis

Statistical analyses was performed using R software, version 3.4.3 (https://www.r-project.org)^55^. The statistical significance was two-sided in this study, with significance level <0.05. Prior to univariate analysis, the fitness of numeric dataset to normal distribution was determined by the Kolmogorov– Smirnov test. Differences in non-parametric data, such as demographic and clinical characteristics and individual perfusion parameters (rCBV and K_trans_), between the PD and PsP groups were assessed using the Mann-Whitney *U* test.

## Supplementary information

Supplementary Information

## Data Availability

The data is available within the Article or the Supplementary Information. The imaging data that support the findings of this study are available from the corresponding author upon request.

## Code availability

For the MR perfusion analysis, we used Nordic ICE, a commercially available processing platform (https://www.nordicneurolab.com/products/nordicICE.html). For volumetric analysis we used 3D Slicer (version 4.3.1, https://www.slicer.org/), an open-source software program widely used for image visualization and segmentation. The pipeline for the radiomic analysis has been introduced in Zinn et al.^20^. For the biostatistical analysis, we used R software (version 3.4.0, R Foundation for Statistical Computing, Vienna, Austria); package MRMRe (version 2.0.7) and C5 decision tree (version 0.1.2) and SVM (version 1.7–0.1)^50–52^.
