# Supplementary material for: Multicenter study demonstrates radiomic features derived from magnetic resonance perfusion images identify pseudoprogression in glioblastoma
Source: Nat Commun. 2019 Jul 18;10:3170. doi: 10.1038/s41467-019-11007-0 (PMC6639324; doi:10.1038/s41467-019-11007-0)
Supplement: Supplementary file 1 — Supplementary Information [file 41467_2019_11007_MOESM1_ESM.pdf]

Supplementary Information (Tables, Figures)

Title: Multicenter study demonstrates radiomic features derived from Magnetic Resonance perfusion images identify pseudoprogression in glioblastoma

Author: Elshafeey et al.

Supplementary Table 1; shows clinical responses according to RANO criteria[10]

Criteria	Complete response	Partial response	Stable disease	Progressive disease
T1 Post Contrast enhancing lesion	None	$\geq$ 50%↓	< 50%↓ but <25%↑	>25%↑*
T2/FLAIR	Stable or↓	Stable or↓	Stable or↓	↑*
New lesion	None	None	None	Present*
Corticosteroids	None	Stable or↓	Stable or↓	NA
Clinical Status	Stable or↑	Stable or↑	Stable or↑	↓*
Requirement for response	All	All	All	Any*

NA, not applicable.
* Progression when this criterion is present

Supplementary Table 2: Demographic and clinical characteristics of 7 MDACC patients with Glioblastoma grade IV (prospective validation set).

Characteristic	Glioblastoma grade IV N=7
Age, years (SD)	57.8571 (14.2995)
Sex, male, N (%)	6 (85.7142)
K_{trans} volume, mm^3 (SD)	4591.56 (6233.76)
rCBV volume, mm^3 (SD)	1396.44 (2245.20)
Surgical type:	
Total resection, N (%)	5 (71.4285)
Sub-total resection, N (%)	2 (28.5714)
Biopsy, N (%)	0
Molecular status:	
MGMT	
Methylated, N (%)	2
Unmethylated, N (%)	2
Non tested, N (%)	3
IDH	
Positive, N (%)	2
Negative, N (%)	4
Non tested, N (%)	0
Radio-therapy time, days (SD)**	60 (52.2857)
Time after RT to PD/PSP, days (SD)***	600.254(643.85)
Chemotherapy treatment (Temozolomide), N (%)	7

rCBV, relative cerebral blood volume; SD, standard deviation; N, Numbers.

Supplementary Table 3: Summary table for patients Perfusion MRI Parameters (e.g TR/TE, slice thickness and Matrix size).

Parameter	Institution		
	MD Anderson	USC	BCM
DCE			
Sequence	GE BS	GE BS	EP/SE
Temporal Resolution	5s	5s	2s
TR/TE, ms	3.597/0.992	6.314/2.228	850/17.4
Slice thickness, mm	4	7	5
Matrix size	256×256	256×186	96×128
Flip angle	15 (pre)<br>2,5,10,20,25 (post)	15 (pre)<br>2,5,10,20,25 (post)	90<br>none
DSC			
Sequence	Single Shot EPI	Single Shot EPI	EP/GE
Temporal Resolution	2s	2s	2s
TR/TE, ms	1500/25	2000/23	2000/13.3
Slice thickness, mm	5	5	5
Matrix size	128×126	128×128	69×128
Flip angle	90	60	60

DCE, dynamic contrast enhanced; TR, repetition time; TE, echo time; DSC, dynamic susceptibility contrast, GE BS, gradient-echo Basic-sequence, EPI, echo-planar imaging, EP/SE, Echo Planar/Spin Echo, EP/GE, Echo Planar/ gradient Echo.

Supplementary Table 4: Perfusion parameter analysis between pseudoprogression and glioblastoma grade IV using K_{trans} and rCBV features showing non-significant results.

Variable	Pseudoprogression N=22	Glioblastoma grade IV N=76	*p*-value *(Mann-Whitney test)*
K_{trans} (SD)	0.2449 (0.3414)	0.2111 (0.3508)	0.858
rCBV (SD)	5.9750 (4.2229)	6.1737 (5.2339)	0.841

rCBV, relative cerebral blood volume.

Supplementary Table 5: Summary of the selected features (60 features); level and Feature name in the K_{trans} map using MRMR method.

Features	Level	Feature name
Ktr_f11	8	Average of Sum of squares: Variance
Ktr_f181	64	Average of Autocorrelation
Ktr_f252	256	Average of Sum average
Ktr_f13	8	Average of Sum variance
Ktr_f121	32	Average of Autocorrelation
Ktr_f191	64	Average of Sum of squares: Variance
Ktr_f61	16	Average of Autocorrelation
Ktr_f253	256	Average of Sum variance
Ktr_f12	8	Average of Sum average
Ktr_f241	256	Average of Autocorrelation
Ktr_fo9	First order	Skewness
Ktr_f132	32	Average of Sum average
Ktr_f251	256	Average of Sum of squares: Variance
Ktr_f72	16	Average of Sum average
Ktr_f193	64	Average of Sum variance
Ktr_f131	32	Average of Sum of squares: Variance
Ktr_f192	64	Average of Sum average
Ktr_f73	16	Average of Sum variance
Ktr_f133	32	Average of Sum variance
Ktr_f5	8	Average of Cluster Shade
Ktr_f130	32	Average of Maximum probability
Ktr_f71	16	Average of Sum of squares: Variance
Ktr_f244	256	Average of Cluster Prominence
Ktr_f210	64	Range of Maximum probability
Ktr_f33	8	Range of Sum variance
Ktr_fo6	First order	Perc5
Ktr_fo10	First order	Kurtosis
Ktr_f250	256	Average of Maximum probability
Ktr_f50	8	Angular Variance of Maximum probability
Ktr_f230	64	Angular Variance of Maximum probability
Ktr_f271	256	Range of Sum of squares: Variance
Ktr_fo3	First order	Mean
Ktr_f125	32	Average of Cluster Shade
Ktr_f270	256	Range of Maximum probability
Ktr_f267	256	Range of Energy
Ktr_f175	32	Angular Variance of Difference variance

Ktr_fo5	First order	Perc1
Ktr_f272	256	Range of Sum average
Ktr_f150	32	Range of Maximum probability
Ktr_f190	64	Average of Maximum probability
Ktr_f116	16	Angular Variance of Difference entropy
Ktr_f65	16	Average of Cluster Shade
Ktr_f290	256	Angular Variance of Maximum probability
Ktr_f30	8	Range of Maximum probability
Ktr_f248	256	Average of Entropy
Ktr_f4	8	Average of Cluster Prominence
Ktr_f249	256	Average of Homogeneity
Ktr_fo1	First order	Minimum
Ktr_f275	256	Range of Difference variance
Ktr_f37	8	Range of Information measure of correlation 1
Ktr_f153	32	Range of Sum variance
Ktr_f147	32	Range of Energy
Ktr_f77	16	Average of Information measure of correlation 1
Ktr_f280	256	Range of Inverse difference moment normalized
Ktr_f245	256	Average of Cluster Shade
Ktr_f207	64	Range of Energy
Ktr_f124	32	Average of Cluster Prominence
Ktr_f288	256	Angular Variance of Entropy
Ktr_f70	16	Average of Maximum probability
Ktr_f151	32	Range of Sum of squares: Variance

Supplementary Table 6: Summary the selected features (160 features); level and Feature name in the rCBV map using MRMR method

Features	Level	Feature name
rCBV_f61	16	Average of Autocorrelation
rCBV_f13	8	Average of Sum variance
rCBV_f241	256	Average of Autocorrelation
rCBV_f72	16	Average of Sum average
rCBV_f121	32	Average of Autocorrelation
rCBV_f181	64	Average of Autocorrelation
rCBV_f11	8	Average of Sum of squares: Variance
rCBV_f14	8	Average of Sum entropy
rCBV_f253	256	Average of Sum variance
rCBV_f12	8	Average of Sum average
rCBV_f133	32	Average of Sum variance
rCBV_f5	8	Average of Cluster Shade
rCBV_f73	16	Average of Sum variance
rCBV_f71	16	Average of Sum of squares: Variance
rCBV_f252	256	Average of Sum average
rCBV_f131	32	Average of Sum of squares: Variance
rCBV_fo5	First Order	Perc 1
rCBV_f193	64	Average of Sum variance
rCBV_f70	16	Average of Maximum probability
rCBV_f251	256	Average of Sum of squares: Variance
rCBV_fo9	First Order	Skewness
rCBV_f192	64	Average of Sum average
rCBV_f138	32	Average of Information measure of correlation 2
rCBV_f191	64	Average of Sum of squares: Variance
rCBV_f10	8	Average of Maximum probability
rCBV_f56	8	Angular Variance of Difference entropy
rCBV_f74	16	Average of Sum entropy
rCBV_f29	8	Range of Homogeneity
rCBV_f249	256	Average of Homogeneity
rCBV_fo1	First Order	Minimum
rCBV_f218	64	Range of Information measure of correlation 2
rCBV_f78	16	Average of Information measure of correlation 2
rCBV_f8	8	Average of Entropy
rCBV_f132	32	Average of Sum average
rCBV_f125	32	Average of Cluster Shade
rCBV_f198	64	Average of Information measure of correlation 2

rCBV_fo10	First Order	Kurtosis
rCBV_f23	8	Range of Correlation
rCBV_f7	8	Average of Energy
rCBV_f18	8	Average of Information measure of correlation 2
rCBV_f30	8	Range of Maximum probability
rCBV_fo6	First Order	Perc 5
rCBV_f129	32	Average of Homogeneity
rCBV_f159	32	Range of Inverse difference normalized (INN)
rCBV_f136	32	Average of Difference entropy
rCBV_f258	256	Average of Information measure of correlation 2
rCBV_f184	64	Average of Cluster Prominence
rCBV_f137	32	Average of Information measure of correlation 1
rCBV_f245	256	Average of Cluster Shade
rCBV_f189	34	Average of Homogeneity
rCBV_fo2	First Order	Maximum
rCBV_f38	8	Range of Information measure of correlation 2
rCBV_f43	8	Angular Variance of Correlation
rCBV_f257	256	Average of Information measure of correlation 1
rCBV_f217	64	Range of Information measure of correlation 1
rCBV_f49	8	Angular Variance of Homogeneity
rCBV_f238	64	Angular Variance of Information measure of correlation 2
rCBV_f277	256	Range of Information measure of correlation 1
rCBV_f197	64	Average of Information measure of correlation 1
rCBV_f36	8	Range of Difference entropy
rCBV_f50	8	Angular Variance of Maximum probability
rCBV_f9	8	Average of Homogeneity
rCBV_f65	16	Average of Cluster Shade
rCBV_f152	32	Range of Sum average
rCBV_f98	16	Range of Information measure of correlation 2
rCBV_f67	16	Average of Energy
rCBV_f146	32	Range of Dissimilarity
rCBV_f178	32	Angular Variance of Information measure of correlation 2
rCBV_f263	256	Range of Correlation
rCBV_f149	32	Range of Homogeneity
rCBV_f124	32	Average of Cluster Prominence
rCBV_f139	32	Average of Inverse difference normalized (INN)
rCBV_f276	256	Range of Difference entropy
rCBV_f77	16	Average of Information measure of correlation 1
rCBV_f196	64	Average of Difference entropy

rCBV_f39	8	Range of Inverse difference normalized (INN)
rCBV_f58	8	Angular Variance of Information measure of correlation 2
rCBV_f69	16	Average of Homogeneity
rCBV_fo8	First Order	Perc99
rCBV_f25	8	Range of Cluster Shade
rCBV_f158	32	Range of Information measure of correlation 2
rCBV_f134	32	Average of Sum entropy
rCBV_f185	64	Average of Cluster Shade
rCBV_f90	16	Range of Maximum probability
rCBV_f59	8	Angular Variance of Inverse difference normalized (INN)
rCBV_f37	8	Range of Information measure of correlation 1
rCBV_f83	16	Range of Correlation
rCBV_f4	8	Average of Cluster Prominence
rCBV_f128	32	Average of Entropy
rCBV_f76	16	verage of Difference entropy
rCBV_f89	16	Range of Homogeneity
rCBV_f31	8	Range of Sum of squares: Variance
rCBV_f27	8	Range of Energy
rCBV_f118	16	Angular Variance of Information measure of correlation 2
rCBV_f219	64	Range of Inverse difference normalized (INN)
rCBV_f68	16	Average of Entropy
rCBV_f96	16	Range of Difference entropy
rCBV_f19	8	Average of Inverse difference normalized (INN)
rCBV_f172	32	Angular Variance of Sum average
rCBV_f103	16	Angular Variance of Correlation
rCBV_f157	32	Range of Information measure of correlation 1
rCBV_f248	256	Average of Entropy
rCBV_f298	256	Angular Variance of Information measure of correlation 2
rCBV_f28	8	Range of Entropy
rCBV_f64	16	Average of Cluster Prominence
rCBV_f100	16	Range of Inverse difference moment normalized
rCBV_fo4	First Order	Standard Deviation
rCBV_f268	256	Range of Entropy
rCBV_f17	8	Average of Information measure of correlation 1
rCBV_f16	8	Average of Difference entropy
rCBV_f179	32	Angular Variance of Inverse difference normalized (INN)
rCBV_f203	64	Range of Correlation
rCBV_f130	32	Average of Maximum probability
rCBV_f156	32	Range of Difference entropy

rCBV_f126	32	Average of Dissimilarity
rCBV_f208	64	Range of Entropy
rCBV_f57	8	Angular Variance of Information measure of correlation 1
rCBV_f24	8	Range of Cluster Prominence
rCBV_f278	256	Range of Information measure of correlation 2
rCBV_f109	16	Angular Variance of Homogeneity
rCBV_f147	32	Range of Energy
rCBV_f244	256	Average of Cluster Prominence
rCBV_f99	16	Range of Inverse difference normalized (INN)
rCBV_f199	64	Average of Inverse difference normalized (INN)
rCBV_f209	64	Range of Homogeneity
rCBV_f110	16	Angular Variance of Maximum probability
rCBV_f154	32	Range of Sum entropy
rCBV_f188	64	Average of Entropy
rCBV_f223	64	Angular Variance of Correlation
rCBV_f148	32	Range of Entropy
rCBV_f85	16	Range of Cluster Shade
rCBV_f214	64	Range of Sum entropy
rCBV_fo7	First Order	Perc 95
rCBV_f239	64	Angular Variance of Inverse difference normalized (INN)
rCBV_f88	16	Range of Entropy
rCBV_f269	256	Range of Homogeneity
rCBV_f186	64	Average of Dissimilarity
rCBV_f216	64	Range of Difference entropy
rCBV_f44	8	Angular Variance of Cluster Prominence
rCBV_f127	32	Average of Energy
rCBV_f279	256	Range of Inverse difference normalized (INN)
rCBV_f256	256	Average of Difference entropy
rCBV_f243	256	Average of Correlation
rCBV_f169	32	Angular Variance of Homogeneity
rCBV_f97	16	Range of Information measure of correlation 1
rCBV_f79	16	Average of Inverse difference normalized (INN)
rCBV_f167	32	Angular Variance of Energy
rCBV_f220	64	Range of Inverse difference moment normalized
rCBV_f151	32	Range of Sum of squares: Variance
rCBV_fo3	First Order	Mean
rCBV_f144	32	Range of Cluster Prominence
rCBV_f150	32	Range of Maximum probability
rCBV_f119	16	Angular Variance of Inverse difference normalized (INN)

rCBV_f254	256	Average of Sum entropy
rCBV_f283	256	Angular Variance of Correlation
rCBV_f274	256	Range of Sum entropy
rCBV_f145	32	Range of Cluster Shade
rCBV_f116	16	Angular Variance of Difference entropy
rCBV_f26	8	Range of Dissimilarity
rCBV_f210	64	Range of Maximum probability

Supplementary Table 7: Summary the selected features (60 features); level and Feature name in the combined K_{trans} and rCBV maps using MRMR method

Features	Level	Feature name
Ktr_f11	8	Average of Sum of squares: Variance
Ktr_f181	64	Average of Autocorrelation
Ktr_f252	256	Average of Sum average
Ktr_f13	8	Average of Sum variance
Ktr_f121	32	Average of Autocorrelation
Ktr_f191	64	Average of Sum of squares: Variance
Ktr_f61	16	Average of Autocorrelation
Ktr_f253	256	Average of Sum variance
Ktr_f12	8	Average of Sum average
Ktr_f241	256	Average of Autocorrelation
Ktr_fo9	First order	Skewness
Ktr_f132	32	Average of Sum average
Ktr_f251	256	Average of Sum of squares: Variance
Ktr_f72	16	Average of Sum average
Ktr_f193	64	Average of Sum variance
Ktr_f131	32	Average of Sum of squares: Variance
Ktr_f192	64	Average of Sum average
Ktr_f73	16	Average of Sum variance
Ktr_f133	32	Average of Sum variance
Ktr_f5	8	Average of Cluster Shade
Ktr_f130	32	Average of Maximum probability
Ktr_f71	16	Average of Sum of squares: Variance
Ktr_f244	256	Average of Cluster Prominence
Ktr_f210	64	Range of Maximum probability
Ktr_f33	8	Range of Sum variance
Ktr_fo6	First order	Perc5
Ktr_fo10	First order	Kurtosis
Ktr_f250	256	Average of Maximum probability
Ktr_f50	8	Angular Variance of Maximum probability
Ktr_f230	64	Angular Variance of Maximum probability
Ktr_f271	256	Range of Sum of squares: Variance
Ktr_fo3	First order	Mean
Ktr_f125	32	Average of Cluster Shade
Ktr_f270	256	Range of Maximum probability
Ktr_f267	256	Range of Energy
Ktr_f175	32	Angular Variance of Difference variance

Ktr_fo5	First order	Perc1
Ktr_f272	256	Range of Sum average
Ktr_f150	32	Range of Maximum probability
Ktr_f190	64	Average of Maximum probability
Ktr_f116	16	Angular Variance of Difference entropy
Ktr_f65	16	Average of Cluster Shade
Ktr_f290	256	Angular Variance of Maximum probability
Ktr_f30	8	Range of Maximum probability
Ktr_f248	256	Average of Entropy
Ktr_f4	8	Average of Cluster Prominence
Ktr_f249	256	Average of Homogeneity
Ktr_fo1	First order	Minimum
rCBV_f203	64	Range of Correlation
Ktr_f280	256	Range of Inverse difference moment normalized
Ktr_f147	32	Range of Energy
Ktr_f77	16	Average of Information measure of correlation 1
Ktr_f153	32	Range of Sum variance
Ktr_f37	8	Range of Information measure of correlation 1
Ktr_f245	256	Average of Cluster Shade
Ktr_f207	64	Range of Energy
Ktr_f124	32	Average of Cluster Prominence
Ktr_f275	256	Range of Difference variance
rCBV_f208	64	Range of Entropy
Ktr_f70	16	Average of Maximum probability

Supplementary Table 8: Table showing the probability of the 7 MDACC new patients from the prospective validation cohort upon prediction using 60 radiomic features by training on a model built with 98 patients for the Ktrans dataset.

Patient ID	Whole dataset		LOOCV		10-fold Cross Validation	
	C5.0 model (%)	SVM model (%)	C5.0 model (%)	SVM model (%)	C5.0 model (%)	SVM model (%)
RPZ-PERF-0000	80.34955	88.45588	77.55102	70.04202	77.55102	97.97734
RPZ-PERF-0001	80.13614	90.41743	77.55102	70.04202	77.55102	98.51650
RPZ-PERF-0002	80.34955	90.63323	77.55102	70.04202	77.55102	98.41448
RPZ-PERF-0004	80.34955	90.52794	77.55102	70.04202	77.55102	98.53143
RPZ-PERF-0005	80.39999	93.43539	77.55102	70.04202	77.55102	98.94774
RPZ-PERF-0006	80.34955	88.13833	77.55102	70.04202	77.55102	97.86269
RPZ-PERF-0008	80.34955	89.01629	77.55102	70.04202	77.55102	98.44284

Supplementary Table 9: Table showing the probability of the 7 MDACC new patients from the prospective validation cohort upon prediction using 160 radiomic features by training on a model built with 98 patients for the rCBV dataset.

Patient ID	Whole dataset		LOOCV		10-fold Cross Validation	
	C5.0 model (%)	SVM model (%)	C5.0 model (%)	SVM model (%)	C5.0 model (%)	SVM model (%)
RPZ-PERF-0000	77.55102	76.12152	77.55102	77.52303	77.55102	79.59756
RPZ-PERF-0001	77.55102	76.12152	77.55102	77.52303	77.55102	79.59756
RPZ-PERF-0002	77.55102	76.12152	77.55102	77.52303	77.55102	79.59756
RPZ-PERF-0004	77.55102	76.12152	77.55102	77.52303	77.55102	79.59756
RPZ-PERF-0005	77.55102	76.12152	77.55102	77.52303	77.55102	79.59756
RPZ-PERF-0006	77.55102	76.12152	77.55102	77.52303	77.55102	79.59756
RPZ-PERF-0008	77.55102	76.12152	77.55102	77.52303	77.55102	79.59756

Supplementary Table 10: Table showing the probability of the 7 MDACC new patients from the prospective validation cohort upon prediction using 60 radiomic features by training on a model built with 98 patients for the combined K_{trans} and rCBV maps.

Patient ID	Whole dataset		LOOCV		10-fold Cross Validation	
	C5.0 model (%)	SVM model (%)	C5.0 model (%)	SVM model (%)	C5.0 model (%)	SVM model (%)
RPZ-PERF-0000	60.63662	87.36898	77.55102	65.27912	77.55102	77.97481
RPZ-PERF-0001	71.19624	87.98548	77.55102	65.27907	77.55102	78.74204
RPZ-PERF-0002	51.15044	87.61988	77.55102	65.27902	77.55102	78.32430
RPZ-PERF-0004	51.09562	88.35771	77.55102	65.27909	77.55102	79.27791
RPZ-PERF-0005	51.03603	85.59855	77.55102	65.27903	77.55102	77.71088
RPZ-PERF-0006	60.68018	88.49370	77.55102	65.27914	77.55102	78.91768
RPZ-PERF-0008	60.63662	88.06298	77.55102	65.27906	77.55102	78.58937

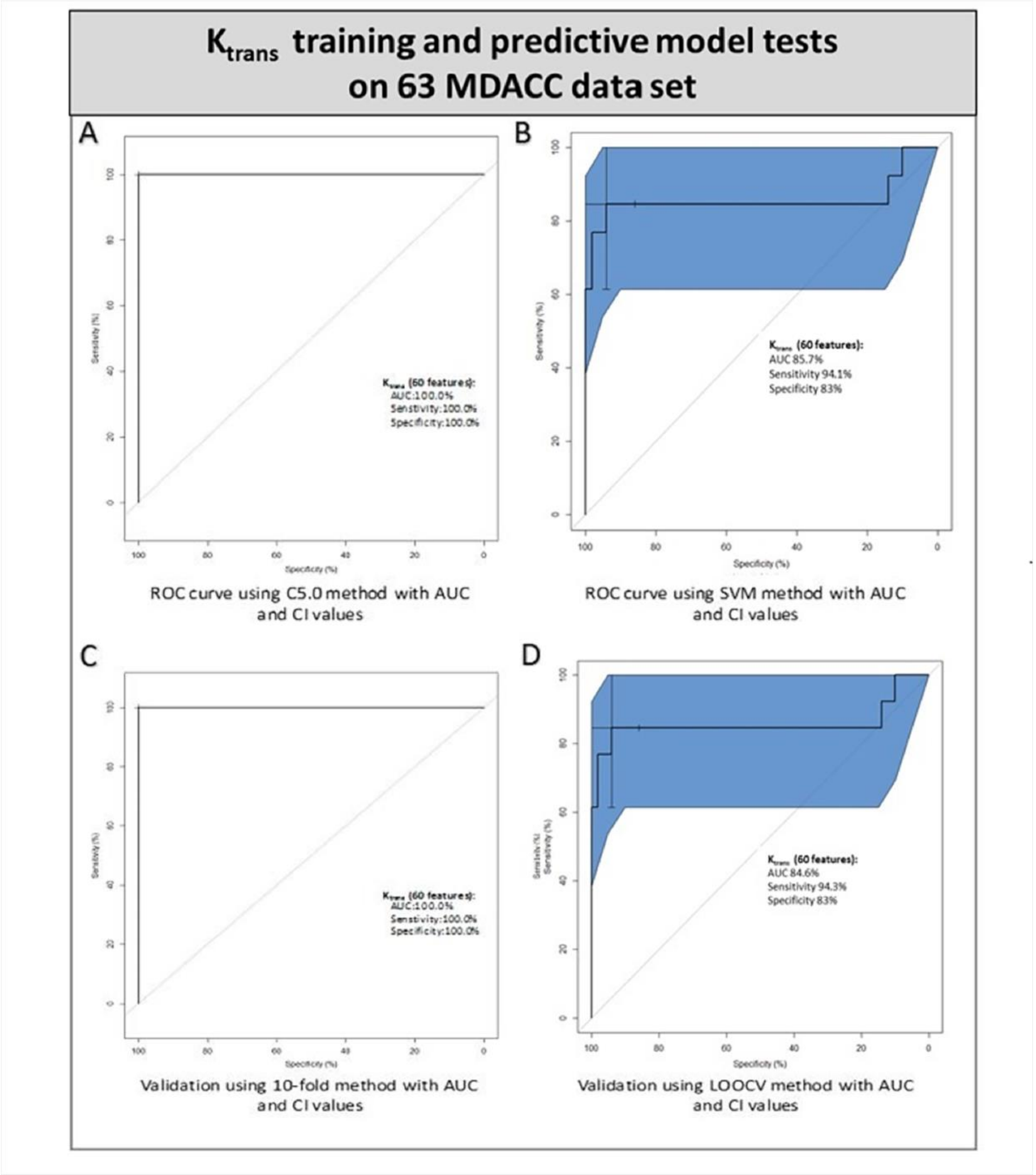

Supplementary figure 1. Model building and evaluation using the selected K_{trans} features (60 features) on 63 MDACC data set. (A, B) ROC curve depicts the predictive model building using C5.0 (*P*-value 4.75e-07) and SVM methods (*P*-value 0.013) respectively. (C, D) 10-fold cross validation ROC curve (*P*-value 4.75e-07) and Leave-One-Out Cross-Validation (LOOCV) ROC curve (*P*-value 0.01246) depicts the performance of the model.

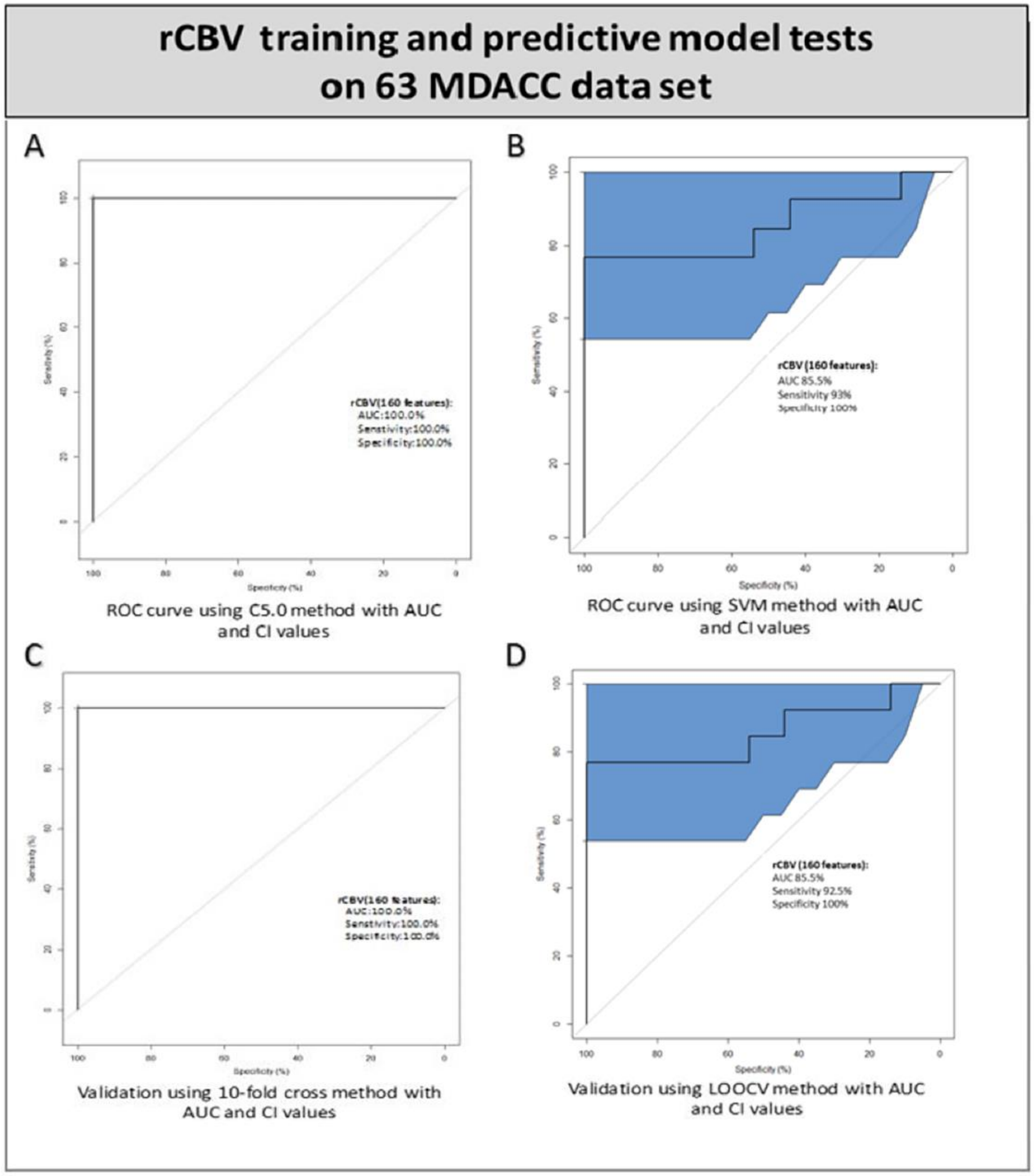

Supplementary figure 2. Model building and evaluation using the selected rCBV features (160 features) on 63 MDACC data set.. (A,B) ROC curve depicts the predictive model building using C5.0 (*P*-value 4.75e-07) and SVM methods (*P*-value 0.043) respectively. (C, D) 10-fold cross validation ROC curve (*P*-value 4.75e-07) and Leave-One-Out Cross-Validation (LOOCV) ROC curve (*P*-value 0.042) depicts the performance of the model.

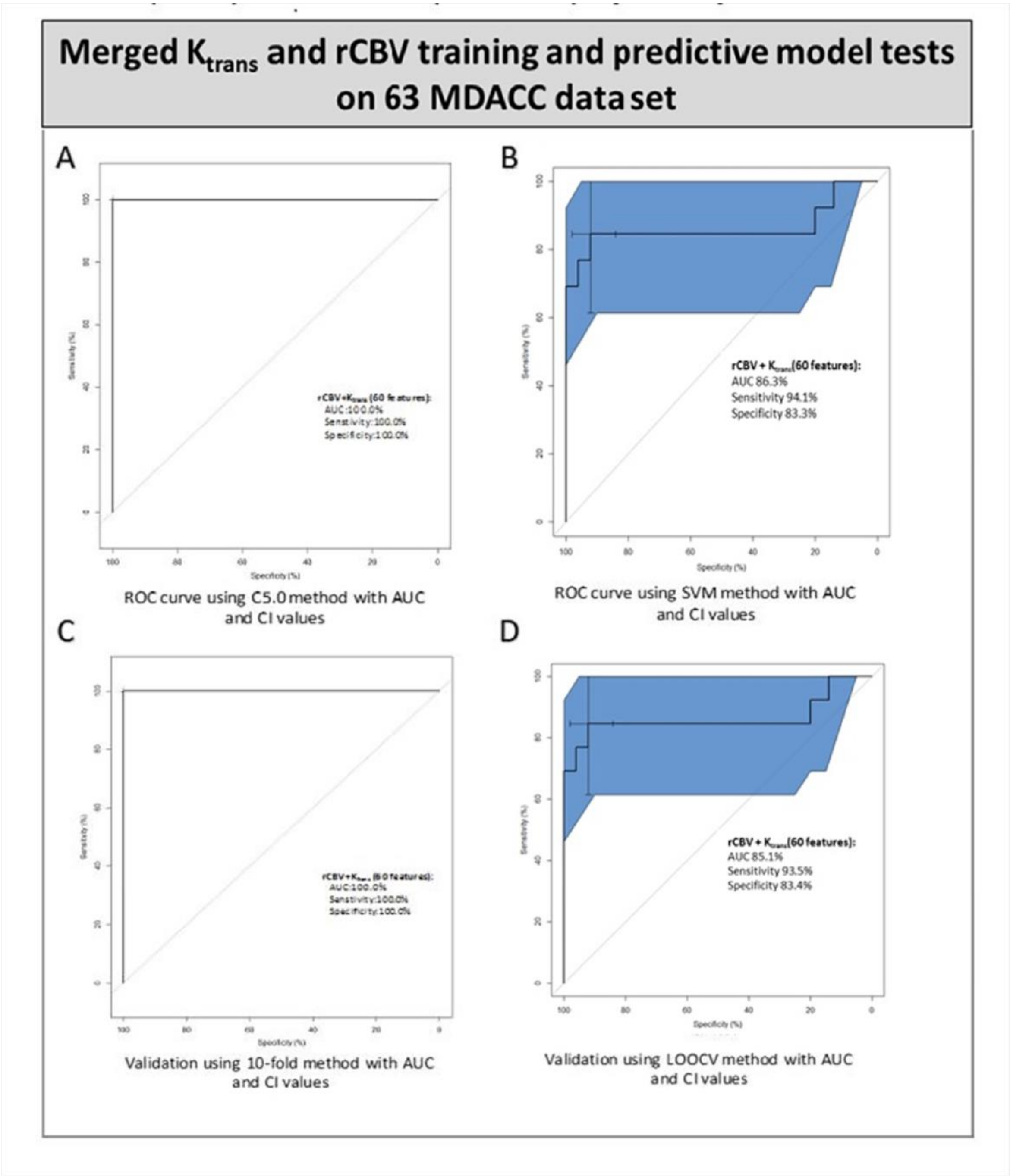

Supplementary figure 3. Model building and evaluation using the selected merged K_{trans} and rCBV features (60 features) on 63 MDACC data set.. (A, B) ROC curve depicts the predictive model building using C5.0 (*P*-value 4.75e-07) and SVM methods (*P*-value 0.0131) respectively. (C, D) 10-fold cross validation ROC curve (*P*-value 4.75e-07) and Leave-One-Out Cross-Validation (LOOCV) ROC curve (*P*-value 0.01246) depicts the performance of the model.

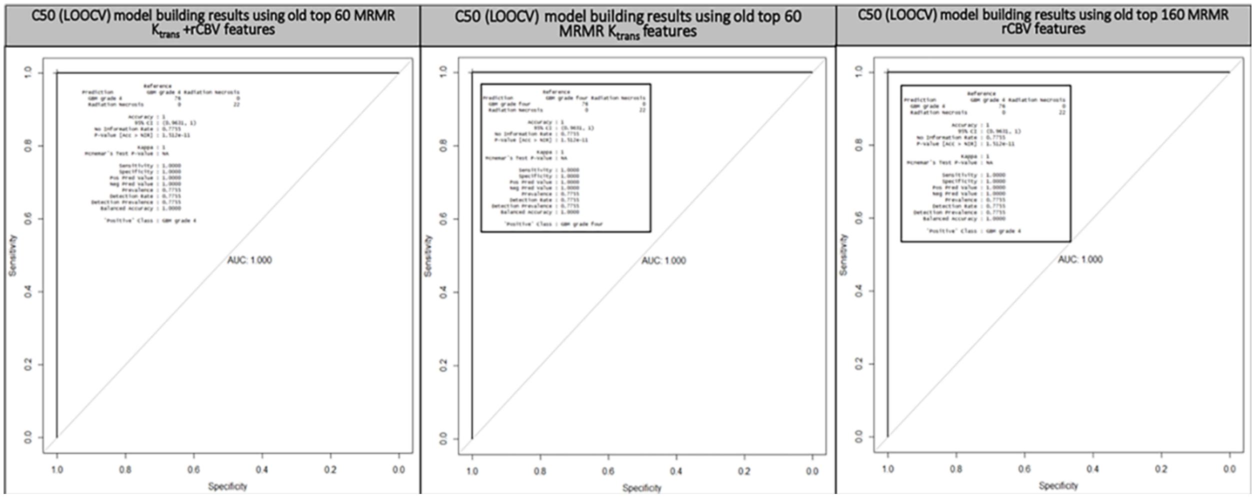

Supplementary figure 4: shows the trial results for applying different cross validation methods on different model building algorithms.
